# Nogo-A exacerbates sepsis-associated encephalopathy by modulating microglial SHP-2/NLRP3 balance and inducing ROS and M1 polarization

**DOI:** 10.17305/bb.2024.10822

**Published:** 2024-08-16

**Authors:** Ying Liu, Lei Guo, Guoan Zhang, Wenjie Sun, Xiaohui Yang, Yingfu Liu

**Affiliations:** 1 Science and Technology Experiment Center, Cangzhou Medical College, Cangzhou, China; 2 Department of Clinical Laboratory, Cangzhou Central Hospital, Cangzhou, China; 3 University Nanobody Application Technology Research and Development Center of Hebei Provice, Cangzhou, China

**Keywords:** Nogo protein, encephalopathy, sepsis, endoplasmic reticulum stress, protein-tyrosine phosphatases, NLRP3 inflammasome, microglia, mitochondria, autophagy, apoptosis

## Abstract

Sepsis, a systemic inflammatory response caused by infection, can lead to sepsis-associated encephalopathy (SAE), characterized by brain dysfunction without direct central nervous system infection. The pathogenesis of SAE involves blood–brain barrier disruption, neuroinflammation, and neuronal death, with neuroinflammation being the core process. Nogo-A, a neurite growth-inhibitory protein in the central nervous system, is not well understood in sepsis. This study explores Nogo-A’s mechanisms in sepsis, focusing on SAE. Using in vivo and in vitro methods, healthy SPF C57BL/6J male mice were divided into Sham, Nogo-A negative control (Nogo-A-NC)-Model, and Nogo-A-KD-Model groups, with sepsis induced by abdominal ligation and puncture. Morris water maze (MWM) tests assessed learning and memory, and brain tissues underwent HE staining, Nissl staining, and western blot analysis. In vitro, Nogo-A gene knockdown models were constructed using BV-2 microglia cells to study inflammation and oxidative stress. Results showed that Nogo-A expression affected learning and memory in septic mice, with knockdown reducing neuronal damage. Bioinformatics analysis suggested Nogo-A may activate ROS to inhibit p-SHP2, activating mitochondrial autophagy and promoting neuronal apoptosis. Western blot results confirmed that Nogo-A affects mitochondrial autophagy and neuronal survival by inhibiting SHP2 and activating ROS. Nogo-A’s role in neuroinflammation and neuroprotection was emphasized, revealing its impact on ER stress, mitochondrial autophagy, and NLRP3 inflammasome activation. This study provides a theoretical basis for SAE treatment, suggesting further multigene and multipathway analyses and validation in clinical samples. Developing gene therapy and drug interventions targeting Nogo-A pathways will offer more effective treatment strategies.

## Introduction

Sepsis is a systemic inflammatory response syndrome caused by infection, which can lead to various complications, one of which is sepsis-associated encephalopathy (SAE). SAE is a broad brain dysfunction associated with severe sepsis, characterized by clinical brain lesions but not caused by direct infection of the central nervous system [1]. During sepsis, acute infections outside the central nervous system, caused by bacteria, viruses, or other pathogens, can lead to a systemic inflammatory response, potentially affecting brain function and leading to the occurrence of SAE. Few studies have indicated that the pathogenesis of SAE includes blood–brain barrier disruption, neuroinflammation, and neuronal death, with neuroinflammation being the core pathological process [2]. SAE often results in cognitive dysfunction, memory impairment, and behavioral abnormalities, reflecting damage to multiple key brain regions and neural circuits [3]. Specifically, the major affected brain regions include the hippocampus, responsible for memory formation and emotional regulation; the parietal cortex, responsible for processing tactile and spatial perception; the temporal cortex, involved in auditory and memory-related functions; and the prefrontal cortex, which is crucial for decision making and executive functions [4, 5]. These damaged regions and their connected neural circuits, such as the prefrontal-hippocampal circuit, affect memory storage, retrieval, and complex cognitive tasks [6, 7]. Although the exact pathogenesis of SAE is not yet fully understood, studies suggest that inflammatory responses and neurotransmitter imbalances may be involved. The real challenge lies in the fact that existing treatments are primarily limited to symptom management and lack effective therapies targeting the underlying mechanisms. Therefore, a deeper understanding of the interactions between these affected brain regions and neural circuits is crucial for discovering new therapeutic targets.

Nogo-A (Rtn4A) is a member of the Rtn4 protein family and is primarily expressed in the central nervous system. As a neurite growth-inhibitory protein, Nogo-A plays an inhibitory role in neural repair and regeneration [8]. Although Nogo-A is mainly expressed in neurons, it is also present in microglia. Microglia are the immune cells of the central nervous system, capable of modulating immune responses and inflammatory processes within the brain. They are key regulators of neuroinflammation and can be activated to either pro-inflammatory (M1) or anti-inflammatory (M2) phenotypes [9]. M1 polarization of microglia activates NF-κB through ROS production, promoting the release of inflammatory factors, such as TNF-α, interleukin 1β (IL-1β), and IL-6, leading to chronic inflammation. Additionally, high levels of ROS may cause apoptosis or necrosis of microglia, affecting the integrity and function of neural networks. Nogo-A may influence neuroinflammation and neuroprotection by modulating the activation state of microglia. It can inhibit the excessive activation of microglia, reduce the release of inflammatory mediators, and thus mitigate the extent of neuronal damage. Nogo-A is widely regarded as an effective inhibitor of axonal regeneration and plasticity in the central nervous system, and its knockout alone can reduce ER stress and prevent cardiomyocyte apoptosis [10]. The mitochondrial-associated ER membrane (MAM) is a special subcellular structure formed by the juxtaposition of ER subdomains with the outer mitochondrial membrane (OMM). MAM has various physiological functions, including calcium ion (Ca2+) transport and signaling, mitochondrial dynamics, apoptosis, autophagy, and regulation of inflammasome formation and activation [11]. However, changes in MAM integrity may lead to adverse effects. Increased calcium ions can lead to increased mitochondrial ROS, which transfers from the ER to the mitochondria, causing mitochondrial damage and releasing mitochondrial components into the cytoplasm as damage-associated molecular patterns, rapidly activating the NLRP3 inflammasome residing in the MAM [12]. NLRP3 is expressed in most tissues but is primarily expressed in microglia. In its inactive state, NLRP3 is located in the ER membrane and cytoplasm, but when activated, NLRP3 and its adaptor protein ASC migrate to the MAM, leading to increased ROS production from damaged mitochondria [13]. SHP2 is a protein tyrosine phosphatase crucial for the regulation of the NLRP3 inflammasome and inflammatory responses [14]. Research indicates that SHP2 is a negative regulator of the NLRP3 inflammasome [15].

Despite extensive research on Nogo-A in the nervous system, its specific mechanisms in sepsis remain unclear. The role of Nogo-A in SAE may be complex, involving both the exacerbation of neuronal damage and potential neuroprotective effects. In sepsis, the impact of the systemic inflammatory response on brain function may lead to the occurrence of SAE. Studies suggest that Nogo-A may regulate sepsis-induced neuroinflammation and damage by inhibiting the proinflammatory phenotype of microglia and ROS production [16]. Besides Nogo-A itself, related pathway proteins such as SHP2 and NLRP3, and other molecules in microglia, may interact with Nogo-A to jointly regulate neuroinflammation and neuronal apoptosis. ER oxidative stress affects MAM function, promoting mitochondrial autophagy and leading to increased ROS. Increased ROS further causes mitochondrial damage and releases damage-associated molecular patterns into the cytoplasm, activating the NLRP3 inflammasome residing in the MAM. Activation of the NLRP3 inflammasome promotes more ROS production, exacerbating mitochondrial damage and inflammatory responses. Current research on Nogo-A and its related pathway proteins in sepsis is still limited and mainly focuses on nervous system damage and regeneration. Future research needs to explore the specific mechanisms of Nogo-A in SAE, including its regulation of microglia activation states, effects on neuroinflammatory processes, and interactions with other pathway proteins. Although preliminary studies have revealed the connection between ROS activation of the NLRP3 inflammasome and the ER-mitochondria interface in inflammation, further research is needed [17].

## Materials and methods

This study systematically investigates the mechanisms of the Nogo-A gene in sepsis through in vivo and in vitro experimental methods. In in vivo experiments, healthy SPF-grade C57BL/6J male mice were randomly divided into three groups after adaptive feeding: Sham group (control group), Nogo-A negative control (Nogo-A-NC)-Model group (Nogo-A gene transfection model group), and Nogo-A-KD-Model group (Nogo-A gene knockout model group). First, brain cells were subjected to Nogo-A gene transfection and conditional knockout operations to ensure effective gene expression. Then, a sepsis model was established using the cecal ligation and puncture (CLP) method. The Morris water maze (MWM) test was used to evaluate the learning and memory abilities of the mice. Brain tissues were collected and processed for HE and Nissl staining to observe the hippocampal brain tissue of the mice and assess the impact of Nogo-A on neuronal morphology. Western blot analysis was also performed to measure the expression levels of Nogo-A, p-SHP2, p-PERK, p-STING, PINK, and Parkin proteins in mouse brain tissue. In in vitro experiments, BV-2 microglial cells were used to establish a Nogo-A gene knockdown model, divided into different treatment groups to study the role of Nogo-A in inflammation and oxidative stress. Cells were divided into seven groups, including the control group (no treatment), Nogo-A-NC-lipopolysaccharide (LPS) group (LPS-induced sepsis), Nogo-A-KD-LPS group (LPS treatment after Nogo-A gene knockdown), and groups with further stimulation of Thapsigargin or PHPS1 for the Nogo-A-NC-LPS and Nogo-A-KD-LPS groups. Specifically, LPS was used to induce inflammation, and Thapsigargin and PHPS1 were used to activate or inhibit specific signaling pathways. Western Blot analysis of Nogo-A, p-SHP2, p-PERK, p-STING, MFN1, NOX2, NOX4, NLRP3, PINK, Parkin, p-TBK1, IL-1β, and other key proteins was conducted to explore the regulatory role of Nogo-A in microglial cell inflammation and oxidative stress and to rule out non-specific effects of different reagents. All data were subjected to rigorous statistical analysis to ensure the accuracy and reproducibility of the experimental results.

### Experimental materials

Twenty-one healthy SPF-grade C57BL/6J male mice, weighing 20 ± 4 g and aged 6–8 weeks, were purchased from Henan Skebes Biotechnology Co., Ltd. (License No.: SCXK (Yue): 2020-0005). A specialized animal laboratory was used to ensure that environmental parameters such as temperature, humidity, and ventilation met standards, avoiding contamination by bacteria or viruses. High-quality feed and sterile water were provided to ensure the mice received adequate nutrition and hydration. Regular health monitoring of the mice, including weight, behavior, and fur color, was conducted to promptly detect and address potential health issues. Environmental quality control was performed regularly, including testing air, water quality, and feed, to ensure compliance with SPF standards. The mice were given adequate acclimatization time before the experiment to help them adapt to the experimental environment and handlers, reducing stress and impact during the experiment.

BV-2 cells were cultured until the logarithmic growth phase (BCRJ Cat# 0356, RRID:CVCL_0182, Shanghai Baililai Biotechnology Co., Ltd.). The cell line was derived from primary mouse microglia infected with a retrovirus carrying the oncogenes v-raf/v-myc, as described by Blasi et al. in 1990 [18]. BV-2 cells were cultured in DMEM high-glucose medium supplemented with 10% FBS, penicillin 100 units/mL (Cat: 15140122, Thermo Fisher Scientific), and streptomycin 100 µg/mL (Cat: 15140148, Thermo Fisher Scientific) in a 37.5% CO_2_ incubator, with media changes every 24 h and subculturing every 48 h to prevent over-confluence or stagnation. Cells were observed under a PZ-XDY-1 inverted microscope to ensure they remained healthy and in the logarithmic growth phase. DMEM high-glucose medium was purchased from Shanghai Yuchun Biotechnology Co., Ltd. The PZ-XDY-1 inverted microscope was purchased from Beijing Pinzhi Chuangsi Precision Instrument Co., Ltd.

Nogo-A plasmids were purchased from Beijing Yiqiao Shenzhou Technology Co., Ltd. (RRID: Addgene_61807) (Cat: 13030-H09E, Sino Biological Inc.).

### Mouse grouping scheme

After one week of adaptive feeding, all mice were randomly divided into three groups (*n* ═ 7 per group). The Sham group was untreated and served as the blank control group. Both the Nogo-A-NC-Model and Nogo-A-KD-Model groups were transfected with the Nogo-A gene. In addition, Nogo-A-KD-Model mice had Nogo-A conditionally knocked out using the Cre-loxP system. After transfection and knockout operations, the Nogo-A-NC-Model and Nogo-A-KD-Model groups underwent sepsis modeling using the CLP method.

Transfection of the Nogo-A gene into mouse brain cells [19, 20]: Mice were first anesthetized and then a viral vector carrying the Nogo-A gene was injected into the brain cells. A stereotaxic apparatus was used to inject the virus into the hippocampus. According to the standard coordinates of the hippocampus, the scalp was disinfected, and a hole was drilled in the skull to expose the brain tissue. The viral vector containing the Nogo-A gene was slowly injected into the hippocampus using a microinjection syringe or glass capillary. After the injection, the site was washed with saline, the skull hole was closed, and the scalp incision was sutured. Postoperative recovery was monitored to ensure no abnormal behavior or signs of infection were observed. A period of 2–4 weeks was allowed to ensure effective expression of the Nogo-A gene.

Construction of Nogo-A conditional knockout mice [21, 22]: First, a gene knockout plan was designed based on the mouse Nogo-A gene sequence, inserting two loxP sites flanking exons 2 and 3 of the Nogo-A gene. Vectors were constructed and introduced into C57BL/6J mouse embryonic stem cells (ES cells) through microinjection and electroporation, and positive clones were selected by G418 screening. Positive ES cells were then implanted into the uteri of pseudopregnant C57BL/6J mice, resulting in Nogo-A gene conditional knockout mice (CKO) with loxP sites. Through backcrossing with C57BL/6J mice, CKO homozygotes were obtained.

CLP method to establish the sepsis model in mice [23, 24]: Mice were first anesthetized with an intraperitoneal injection of pentobarbital sodium (50 mg/kg) and placed on a temperature-controlled blanket (507220F, Harvard Apparatus) to maintain body temperature at 36.5 ^∘^C. A longitudinal incision was made along the midline of the abdomen to expose the cecum. The cecum was ligated 1 cm from the tip with sterile 4-0 silk sutures and punctured with a sterile needle to release a small amount of intestinal contents, ensuring a patent perforation. The cecum was then returned to the abdomen, and the surgical incision was closed in layers.

After the experiment, mice were euthanized under gas anesthesia with 20% isoflurane, and brain tissue samples were collected for subsequent analysis. The study was reviewed and approved by the Ethics Committee of Cangzhou Medical College. All experiments were conducted in accordance with relevant guidelines and regulations. The authors of this study followed the ARRIVE guidelines.

### MWM test

The MWM test was used to evaluate the learning and memory abilities of all mice. During this test, each mouse was placed in a Morris mouse temperature-controlled swimming pool (24 ^∘^C–26 ^∘^C), a black stainless steel drum (diameter 1500 mm, height 500 mm) [25]. The water in the pool was made opaque by adding nontoxic black ink and was divided into four quadrants: I, II, III, and IV. A circular platform with a diameter of 12 cm and height of 30 cm was submerged 2 cm below the water surface in quadrant IV. Reference cues and room lights were maintained around the pool. Mice were placed in the pool from each quadrant facing the wall, and the time taken for them to climb onto the hidden platform (escape latency) was recorded. If the platform was not found within 60 s, the mouse was guided to the platform for 20 s, with the escape latency recorded as 60 s. A spatial probe test was performed the next day after the place navigation test. Mice were placed in the pool twice from the opposite quadrant and allowed to explore freely for 120 s. The MWM video analysis system tracked and recorded the number of times the mouse crossed the hidden platform. At the end of each test, mice were dried under an Exo Terra infrared heat lamp cage for 1–2 min before returning to their normal cage. The Morris mouse temperature-controlled swimming pool was purchased from Anhui Zhenghua Biological Instrument and Equipment Co., Ltd. The Exo Terra infrared heat lamp was purchased from Shuyang Lichong Xinyi Electronic Commerce Co., Ltd.

### Collection of mouse brain tissue

One hour after the MWM test, mice were placed in a precooled balanced solution and decapitated under deep anesthesia. Brain tissue was removed and divided into two parts for processing. One part was used for HE and Nissl staining. The brain tissue was immediately placed in a dish containing 4% neutral buffered formalin for fixation for 4–24 h to ensure tissue structure stability. The fixed brain tissue was dehydrated and embedded in paraffin, and 4–6 µm thick sections were prepared. Sections could be used for staining and stored at room temperature, or mounted with a mounting medium to protect the staining effect. The other part was used for western blot analysis. Small pieces of fixed brain tissue were cut, and protein was extracted using a lysis buffer containing protease and phosphatase inhibitors. Extracted proteins were aliquoted and stored at --80 ^∘^C to maintain stability.

### HE staining

Five tissue sections from different positions of each mouse were taken, with a total of 35 sections per group. Paraffin sections were dissolved in xylene and dewaxing was performed using an ethanol gradient. The dewaxed sections were placed in a hematoxylin staining solution, typically a diluted hematoxylin solution. Staining time was adjusted as needed, generally 2–10 min, as excessive staining time may lead to over-staining [26]. The sections were then rinsed with distilled water to remove excess dye. Next, the sections were placed in acidic alcohol or acidic solutions for acid washing to remove excess hematoxylin dye. The sections were placed in a blueing agent for 1–5 min, such as acidic alcohol or acidic agarose, to enhance the hematoxylin staining effect. The sections were again rinsed with distilled water to remove excess dye. The sections were stained with eosin dye, and thoroughly rinsed with distilled water to remove excess dye. The sections were dehydrated through an ethanol gradient, immersed in a clearing agent to make them transparent and ultimately mounted. The sections were mounted and observed under an Olympus BX53 optical microscope. Grouping was the same as for the MWM.

### Nissl staining

For each mouse, three tissue slices from different brain regions were collected, totaling 21 slices per group. The paraffin sections were deparaffinized with xylene, dehydrated with gradient alcohol, and stained at room temperature for 20 min with 1% Cresyl Violet (Cat: N21480, Thermo Fisher Scientific). The sections were then washed in distilled water and differentiated with 70% alcohol. Subsequently, they were immersed in gradient alcohol and xylene for 2 min each. Nissl cell counts were performed using an Olympus BX53 light microscope. The grouping was the same as that for MWM. The Olympus BX53 light microscope was purchased from Qingdao Topscience Technology Development Co., Ltd.

### Hippocampal neuron ultrastructure

Three mice from each group were randomly selected, and two hippocampal samples per mouse were taken, totaling six hippocampal samples per group for Transmission Electron Microscope (TEM) observation. The hippocampal tissue (approximately 1 mm × 1 mm × 3 mm) was fixed with 2.5% Glutaraldehyde (Cat: P1126, Beijing Solarbio Science & Technology Co., Ltd) and 1% Osmium Tetroxide (OsO_ImEquation1_), then dehydrated with a series of ethanol solutions, embedded in epoxy resin, and double-stained with Uranyl Acetate and Lead Citrate [27, 28]. The ultrastructure of hippocampal neurons was observed using a TEM (Talos F200X S, Ningbo Zhenjing Optical Instrument Co., Ltd).

### Bioinformatics analysis

Using the GEO database (http://www.ncbi.nlm.nih.gov/geo/) on the NCBI platform, the search condition is set to “SAE.” The relevant mRNA gene expression dataset GSE135838 (based on GPL20301 probe platform Illumina HiSeq 4000 [Homo sapiens]) is downloaded and normalized using R language (version 4.1.0) and related packages [29–31]. Differential expression analysis is performed using the Limma R package to obtain differential genes (DEGs), and volcano plots and heatmaps of DEGs are generated. The threshold for DEGs is generally set at |logFC| > 1, *P* < 0.05.

Online analysis of DEGs is conducted using the DAVID (Database for Annotation, Visualization and Integrated Discovery) bioinformatics resource database with Homo sapiens as the background. Gene Ontology (GO) and Kyoto Encyclopedia of Genes and Genomes (KEGG) enrichment analyses are performed to identify gene clusters and pathways with biological characteristic differences among DEGs. The R language (version 4.1.0) ggplot2 package is used to plot bar charts and chord diagrams for GO upregulated and downregulated gene enrichment analysis, and scatter plots for KEGG enrichment analysis.

To explore gene co-expression properties in the dataset, single-gene co-expression scatter plots for STING1 (Stimulator Of Interferon Response CGAMP Interactor 1), TBK1 (TANK Binding Kinase 1), ROS1 (ROS Proto-Oncogene 1), PTPN11 (Protein Tyrosine Phosphatase Non-Receptor Type 11), IRF3 (Interferon Regulatory Factor 3), NLRP3 (NLR Family Pyrin Domain Containing 3), and RTN4 (Reticulon 4) are plotted using the ggstatsplot package in R language (version 4.1.0).

### BV-2 cell grouping and treatment

Random samples of BV-2 cells during the logarithmic growth phase from section 1.1 are divided into seven groups, with each group containing 2×10^6^ cells, placed into 24-well plates. Group ① (Control Group) is left untreated; Group ③ (Nogo-A-KD-LPS) consists of Nogo-A gene knockdown cells; Group ② (Nogo-A-NC-LPS) adds an equal volume of physiological saline as Group ③, and both Groups ② and ③ use the same volume of LPS to induce sepsis models. Groups ④ (Nogo-A-NC-LPS-Thapsigargin) and ⑥ (Nogo-A-NC-LPS-PHPS1) receive the same physiological saline and LPS treatment as Group ②. Groups ⑤ (Nogo-A-KD-LPS-Thapsigargin) and ⑦ (Nogo-A-KD-LPS-PHPS1) undergo the same Nogo-A gene knockdown and LPS treatment as Group ③. Then, Groups ④ and ⑤ are treated with an equal amount of ERS agonist Thapsigargin; Groups ⑥ and ⑦ are treated with an equal amount of SHP2 inhibitor PHPS1. The model construction details are as follows:

Constructing Nogo-A Gene Knockdown Cell Model: Design shRNA sequences targeting the Nogo-A gene, clone these sequences into the pLKO.1 vector (Cat: KM1022515, Wenzhou Kemiou Biotechnology Co., Ltd), and transfect the plasmid containing shRNA expression vectors into BV2 cells using liposomes to initiate shRNA expression. After transfection, cells are selected with puromycin to isolate those that have successfully integrated the shRNA expression vector and can stably express shRNA, achieving Nogo-A gene knockdown.

LPS-Induced Sepsis Model: BV-2 cells are cultured in DMEM medium with 10% FBS (Cat: 11011-8611, Beijing Solarbio Science & Technology Co., Ltd) and antibiotics (100 U/mL penicillin and 100 µg/mL streptomycin) at 37 ^∘^C in a 5% CO_2_ humidified atmosphere. When cells reach 70%–80% confluence, LPS is added at a concentration of 1 µg/mL for 12 h to induce an inflammatory response (LPS, Cat: L8880, Beijing Solarbio Science & Technology Co., Ltd).

Thapsigargin Stimulation Procedure: Prepare a Thapsigargin solution at a concentration of 0.353 nM, dissolve it in PBS, and filter sterilize. Seed cells in culture plates and ensure they are in the logarithmic growth phase and in good condition before Thapsigargin stimulation. Add the Thapsigargin solution to the cell culture medium and gently mix. Incubate the treated cells for 24 h. Thapsigargin (Cat: HY-13433, MedChemExpress).

PHPS1 Stimulation Procedure: Prepare a PHPS1 solution at a concentration of 5 µM, add it to the cell culture medium, and gently mix. Incubate the treated cells for 24 h and observe the effect of PHPS1 on the cells. PHPS1 (Cat: HY-112368, MedChemExpress).

### Western blotting

Seven mice per group, with two brain tissue blocks per mouse for protein extraction, totaling 14 brain tissue blocks per group for in vivo western blotting. After removing brain tissue from the --80 ^∘^C freezer, thaw and mix uniformly to ensure complete protein dissolution. Use a lysis buffer with protease and phosphatase inhibitors for protein extraction, then centrifuge to obtain total protein from the supernatant. Separate proteins using SDS-PAGE and transfer them to a PVDF membrane. Block the membrane at room temperature for 2 h to reduce non-specific binding. Incubate membrane proteins with primary antibodies overnight at 4 ^∘^C for specific binding. Next, add secondary antibodies and incubate at 37 ^∘^C for 90 min. After incubation, detect proteins using chemiluminescence and measure band density with Image J software. Quantify the expression levels of Nogo-A, p-SHP2, p-PERK, p-STING, PINK, and Parkin proteins in Sham, Nogo-A-NC-Model, and Nogo-A-KD-Model groups.

After culturing and treating BV2 cells from different experimental groups, harvest cells, lyse with lysis buffer, centrifuge to remove cell debris, and measure protein concentration. Separate proteins using SDS-PAGE and transfer them to PVDF or nitrocellulose membranes. Block the membranes to prevent non-specific binding, incubate with specific primary antibodies (e.g., Nogo-A and p-PERK), wash to remove unbound primary antibodies, and incubate with HRP-conjugated secondary antibodies. Detect proteins using chemiluminescence. Finally, analyze the band signals on membranes to compare protein expression levels of Nogo-A, p-PERK, MFN1, NOX2, NOX4, NLRP3, p-SHP2, PINK, Parkin, p-STING, p-TBK1, and IL-1β across seven groups to analyze the impact of Nogo-A on protein expression in BV2 cells through the regulation of oxidative stress/SHP-2/NLRP3.

### Ethical statement

This study has been reviewed and approved by Ethics Committee of Cangzhou University of Higher Medical Sciences (SYDW-2023002).

### Statistical analysis

Data are presented as mean ± SD. Comparisons between two groups were conducted using the independent samples *t*-test or Mann–Whitney *U* test; one-way ANOVA was used for comparisons among three or more groups. Bioinformatics analysis included differential expression analysis using the Limma package, GO and KEGG enrichment analysis using the DAVID database, and generation of relevant plots. All plots were created using GraphPad Prism or R, and all analyses were performed using SPSS or R. * denotes *P* < 0.05, ** denotes *P* < 0.01, with *P* < 0.05 considered statistically significant.

**Figure 1.**
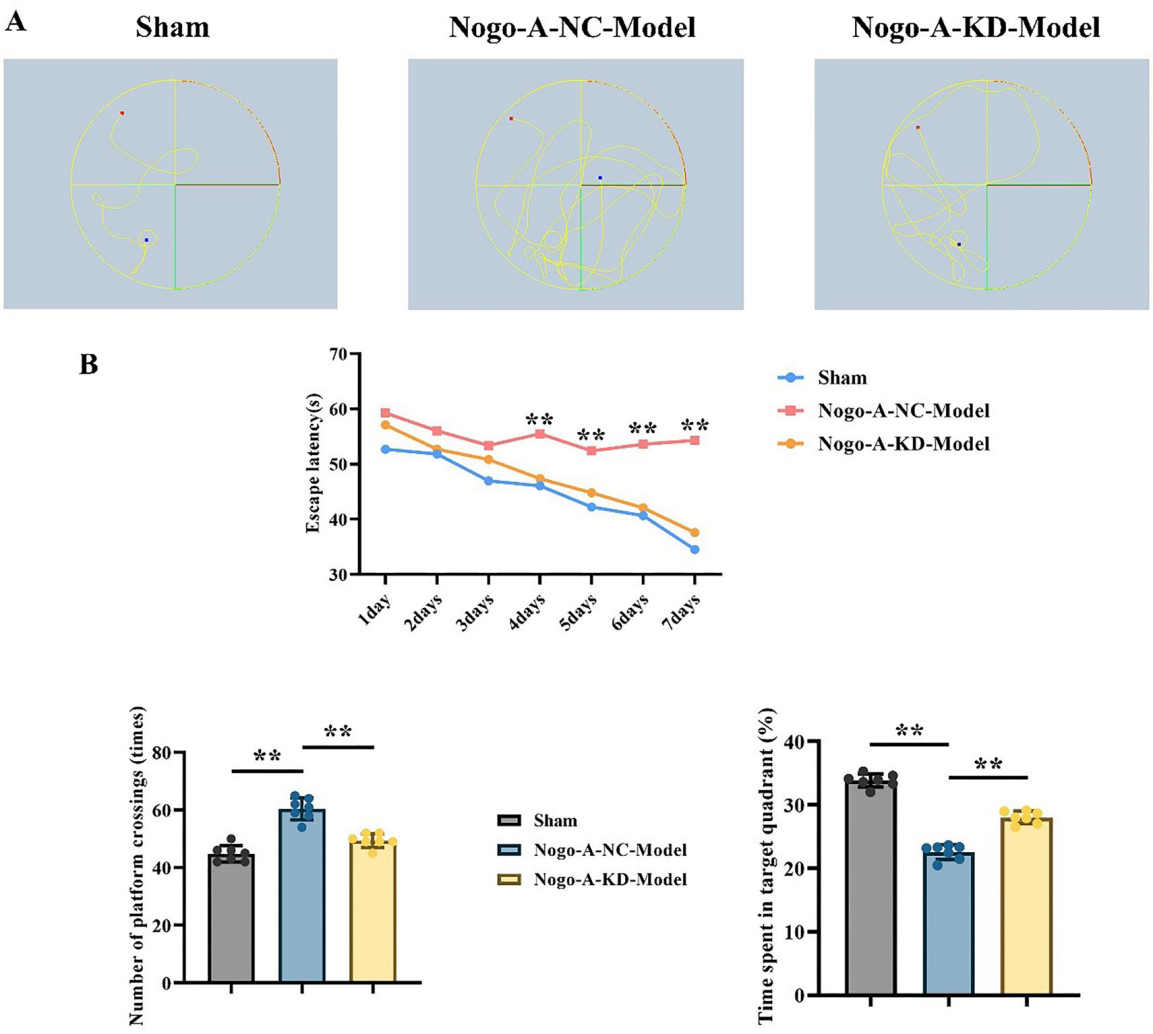
**The effect of Nogo-A expression in septic mouse models on learning and memory in the Morris water maze test (*n* ═ 7).** (A) Swimming trajectories of mice in the Morris water maze test. Left: The Sham group, with relatively simple trajectories and shorter time to find the platform. Middle: The Nogo-A-NC-Model group, with more complex trajectories and longer time to find the platform. Right: The Nogo-A-KD-Model group, with relatively shorter trajectories and shorter time to find the platform; (B) Escape latency curve, number of platform crossings, and time spent in the target quadrant in the Morris water maze. The line graph shows that the Sham group had a significantly shorter escape latency throughout the test, while the Nogo-A-NC-Model group had a significantly longer escape latency compared to the control group, and the Nogo-A-KD-Model group had a significantly shorter escape latency. The bar graph illustrates the number of platform crossings and time spent in the target quadrant for each group. Mice in the Sham group had significantly more platform crossings and spent more time in the target quadrant compared to the Nogo-A-NC-Model group, while the Nogo-A-KD-Model group performed significantly better in both metrics compared to the Nogo-A-NC-Model group. Note: Data are presented as mean ± standard deviation (Mean ± SD), **indicates *P* < 0.01. Nogo-A-NC: Nogo-A negative control.

## Results

In the MWM test, the expression or absence of Nogo-A significantly affected the learning and memory abilities of septic model mice. The spatial exploration test results showed that mice in the Nogo-A knockdown group had more complex swimming trajectories, longer escape latencies, fewer crossings of the platform location, and spent less time in the target quadrant, indicating worse performance compared to the Nogo-A-NC group and the blank control group. Histological observations using HE and Nissl staining, as well as transmission electron microscopy, revealed that neurons in the Nogo-A-NC group were most severely damaged, with disorganized neuron arrangement, atrophy, and degenerative changes. In contrast, the neuronal damage in the Nogo-A knockdown group was alleviated and approached the normal state of the blank control group. Bioinformatics analysis suggested that Nogo-A might activate ROS to inhibit p-SHP2, thereby activating mitochondrial autophagy and promoting neuronal apoptosis. In vivo western blot results indicated that the expressions of Nogo-A, PINK, Parkin, p-PERK, and p-STING were significantly increased in the Nogo-A-NC group, while the expression of p-SHP2 was significantly decreased. After Nogo-A knockdown, the expressions of SHP2, PINK, and Parkin increased, while the expressions of Nogo-A, p-PERK, and p-STING decreased. In vitro western blot experiments further showed that in the septic model, elevated Nogo-A expression inhibited SHP2 expression, leading to increased expression of various proteins and endoplasmic reticulum oxidative stress, whereas Nogo-A knockdown improved the abnormalities in protein expression. In summary, Nogo-A affects mitochondrial autophagy and neuronal survival by inhibiting SHP2 and activating ROS, as validated by both in vivo and in vitro experiments.

### Nogo-A reduces mouse intelligence

The MWM test was used to evaluate whether the expression or absence of Nogo-A affects learning and memory abilities in mice with a septic model. In the spatial exploration test, swimming trajectories (Figure 1A), time spent in the target quadrant (Figure 1B), and the number of crossings of the original platform location (Figure 1C) were assessed. Mice in the Sham group exhibited simpler swimming trajectories and shorter times to find the platform, whereas mice in the Nogo-A-NC-Model and Nogo-A-KD-Model groups performed worse. Compared to the Nogo-A-NC-Model group, mice in the Nogo-A-KD-Model group showed reduced escape latencies, fewer crossings of the platform location, and less time spent in the target quadrant.

### Nogo-A damages the CA region of the mouse hippocampus

**Figure 2.**
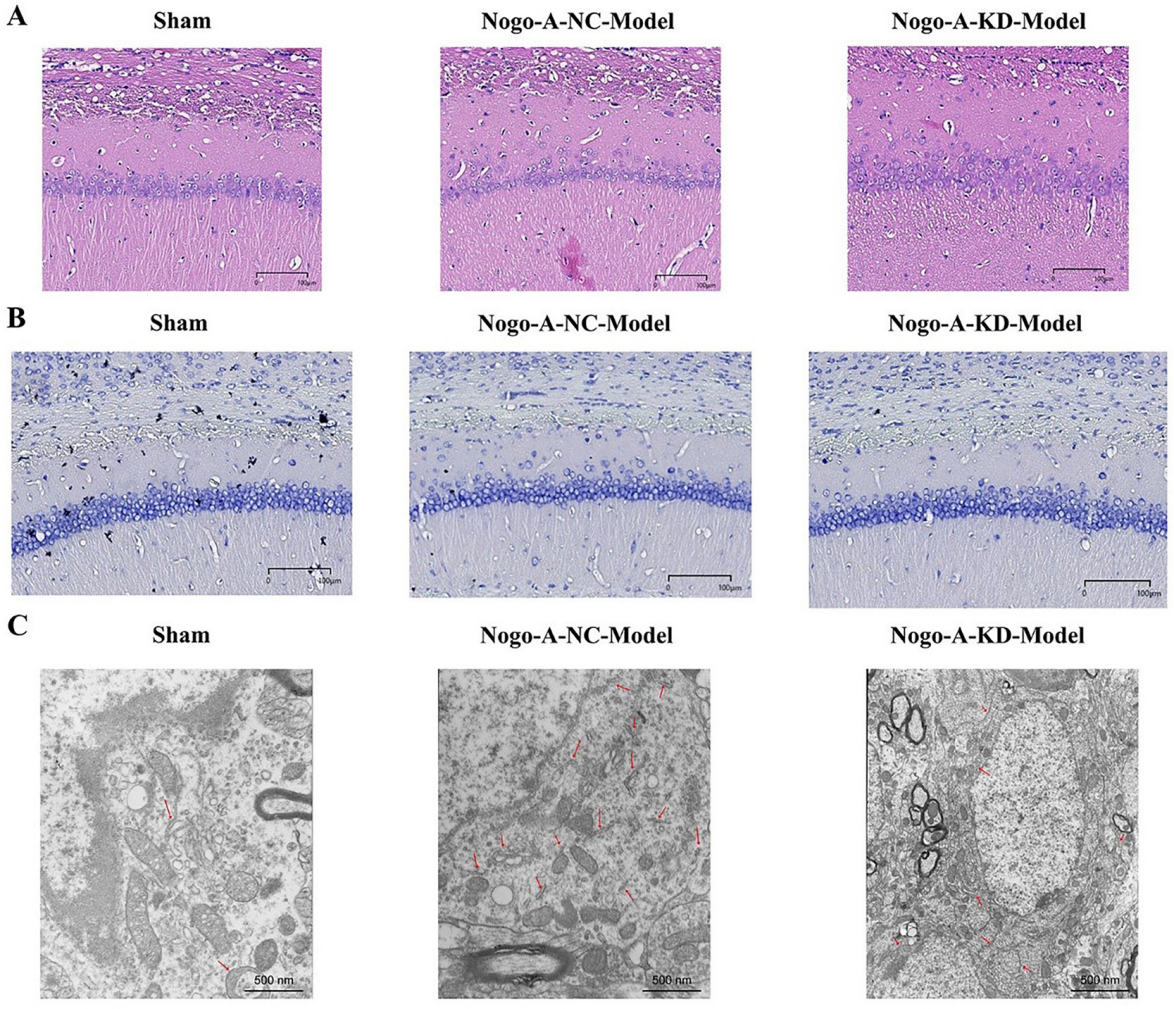
**Histological changes in hippocampal slices of mice under different treatment conditions.** (A) Hematoxylin–eosin (H&E) staining of hippocampal slices (*n* ═ 35). From left to right: Sham group, Nogo-A-NC-Model group, Nogo-A-KD-Model group. The Nogo-A-KD-Model group showed significant neuronal damage compared to the Sham group (*P* < 0.01). (B) Nissl staining of hippocampal slices (*n* ═ 21). From left to right: Sham group, Nogo-A-NC-Model group, Nogo-A-KD-Model group. The Nogo-A-KD-Model group had a significant reduction in Nissl-positive cells compared to the Nogo-A-NC-Model group (*P* < 0.01). (C) Transmission electron microscopy observation of neuronal tissue in mouse brains (*n* ═ 6). From left to right: Sham group, Nogo-A-NC-Model group, Nogo-A-KD-Model group. The Sham group: Clear cellular structure with normal organelles like mitochondria and ER, with no significant damage. The Nogo-A-NC-Model group: Blurred cell structure with swollen and damaged organelles (e.g., mitochondria), indicating significant cellular damage and stress response. The Nogo-A-KD-Model group: Relatively intact cellular structure, with reduced organelle damage compared to the Nogo-A-NC-Model group, suggesting a protective effect of Nogo-A gene knockdown. Red arrows indicate damaged areas of organelles (*P* < 0.01). Note: Data are presented as mean ± standard deviation (Mean ± SD), **indicates *P* < 0.01. Nogo-A-NC: Nogo-A negative control.

Histological results are as follows: In the Sham group, neurons in the cortical region of the brain were neatly arranged, with normal morphology, clear boundaries, round nuclei, and prominent nucleoli. The Nogo-A-NC-Model group showed disorganized neuron arrangement, contraction, and degenerative changes, with unclear nuclear boundaries, missing nuclei, and interstitial edema. Compared to the Nogo-A-NC-Model group, the Nogo-A-KD-Model group exhibited lower degrees of neuron atrophy, degenerative changes, and interstitial edema in the cortical region of the brain (Figure 2A).

**Figure 3.**
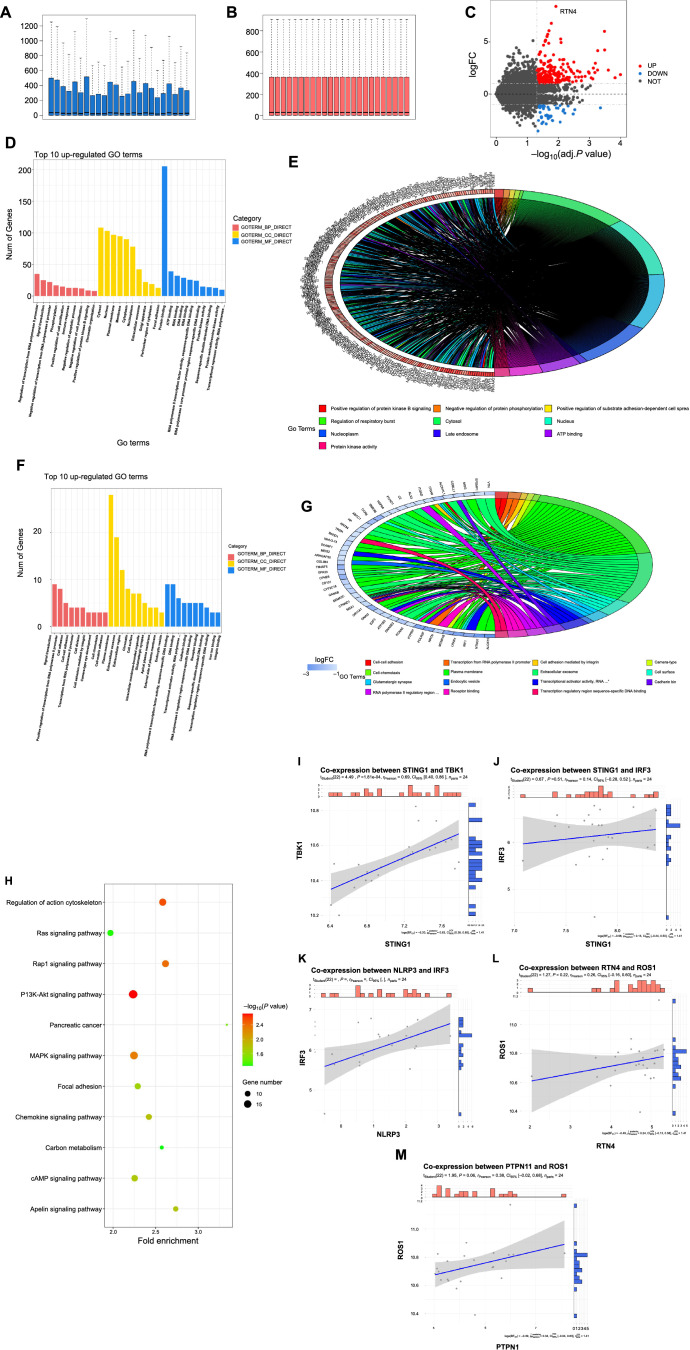
**Gene expression differences, functional enrichment, and co-expression analysis.** The GSE135838 dataset includes 12 inflammatory samples and 12 control samples (see Figure A). After preprocessing and calibration (see Figure B), DESeq2 analysis identified the significantly upregulated gene Nogo-A (RTN4) (see Figure C). GO enrichment analysis: GOplot and ggplot2 were used to create bar charts ranking the top 10 GO pathways for upregulated differentially expressed genes, including biological processes (BP), cellular components (CC), and molecular functions (MF). The results show significant enrichment in biological processes such as “regulation of transcription from RNA polymerase II promoter,” “signal transduction,” and “negative regulation of transcription from RNA polymerase II promoter” (see Figure D). A chord diagram of GO pathways for upregulated genes was created to show relationships between these biological processes (see Figure E). For downregulated genes, bar charts of the top 10 GO pathways were plotted, including BP, CC, and MF, showing significant enrichment in biological processes such as “signal transduction,” “positive regulation of transcription from RNA polymerase II promoter,” and “cell adhesion” (see Figure F). A chord diagram of GO pathways for downregulated genes was created to show the connections between these biological processes (see Figure G). KEGG pathway analysis: KEGG bubble plots illustrate enrichment in signaling pathways such as the “Ras signaling pathway,” “PI3K-Akt signaling pathway,” and “MAPK signaling pathway” (see Figure H). Gene co-expression analysis: Scatter plots were created using the ggstatsplot package to show co-expression relationships between STING1 and TBK1, with Pearson correlation coefficients (see Figure I), between STING1 and IRF3 (see Figure J), between IRF3 and ROS1 (see Figure K), between RTN4 and ROS1 (see Figure L), and between PTPN11 and ROS1 (see Figure M). Different colors or markers indicate gene expression levels, and mean lines show average expression levels. Analysis results indicate that STING1 promotes the expression of TBK1 and IRF3, while IRF3, RTN4, and PTPN11 promote ROS1 expression. GO: Gene Ontology; KEGG: Kyoto Encyclopedia of Genes and Genomes; MAPK: Mitogen-activated protein kinase.

Nissl staining revealed pathological changes in the CA region of the hippocampus across all groups: Neurons in the Sham group appeared normal, orderly, round, and clear, with prominent nucleoli. In the Nogo-A-NC-Model group, neurons showed degenerative changes and necrosis, with neuronal cell atrophy, increased nuclear pigment deposition, reduced or absent nuclei, and enlarged cell spaces observed under the microscope. Compared to the Nogo-A-NC-Model group, the Nogo-A-KD-Model group had reduced neuron atrophy and nuclear fragmentation (Figure 2B).

TEM observations of mouse brain tissue revealed significant differences in cell structure across groups. In the Sham group, cells had clear structures with the normal morphology of organelles, such as mitochondria and endoplasmic reticulum, indicating good cell health with no apparent damage. In the Nogo-A-NC-Model group, cells exhibited blurred and disorganized structures with clear signs of organelle damage, including swollen mitochondria and membrane structure disruption, suggesting significant cellular stress or damage. In the Nogo-A-KD-Model group, although some structural issues with the endoplasmic reticulum and mitochondria were still observed, the overall cell structure was more intact compared to the Nogo-A-NC-Model group, indicating that Nogo-A gene knockdown may have some protective effects, alleviating cellular damage and stress responses (Figure 2C).

### Bioinformatics analysis based on the GSE135838 dataset reveals expression characteristics, GO pathway enrichment, and KEGG pathway analysis of inflammation-related gene Nogo-A

The GSE135838 dataset contains 12 inflammation group datasets and 12 control group datasets (Figure 3A). After preprocessing and normalization (Figure 3B), DESeq2 analysis identified the significantly upregulated gene Nogo-A (RTN4) (Figure 3C).

GOplot and ggplot2 packages in R language were used to draw bar plots of the top 10 upregulated differentially expressed genes in the BP, CC, and MF GO pathways (Figure 3D) and chord diagrams of upregulated GO pathways (Figure 3E), showing enrichment in biological processes such as regulation of transcription from RNA polymerase II promoter, signal transduction, and negative regulation of transcription from RNA polymerase II promoter. Bar plots of the top ten downregulated differentially expressed genes in BP, CC, and MF GO pathways (Figure 3F) and chord diagrams of downregulated GO pathways (Figure 3G) indicated enrichment in processes, such as signal transduction, positive regulation of transcription from RNA polymerase II promoter, and cell adhesion. KEGG enrichment analysis with a KEGG bubble plot showed enrichment in pathways, such as the Ras signaling pathway, PI3K-Akt signaling pathway, and mitogen-activated protein kinase (MAPK) signaling pathway (Figure 3H).

Using the ggstatsplot package in R (version 4.1.0), scatter plots of co-expression relationships were drawn for STING1 and TBK1 (Figure 3I), STING1 and IRF3 (Figure 3J), IRF3 and ROS1 (Figure 3K), RTN4 and ROS1 (Figure 3L), and PTPN11 and ROS1 (Figure 3M), and Pearson correlation coefficients were calculated. Different colors or markers were used to represent the expression levels of different genes, and average value lines were used to visually display average expression levels. STING1 was found to promote the expression of TBK1, IRF3, and ROS1, while RTN4 and PTPN11 also promoted ROS1 expression.

### In vivo western blot shows Nogo-A indirectly inhibits p-SHP2 expression

**Figure 4.**
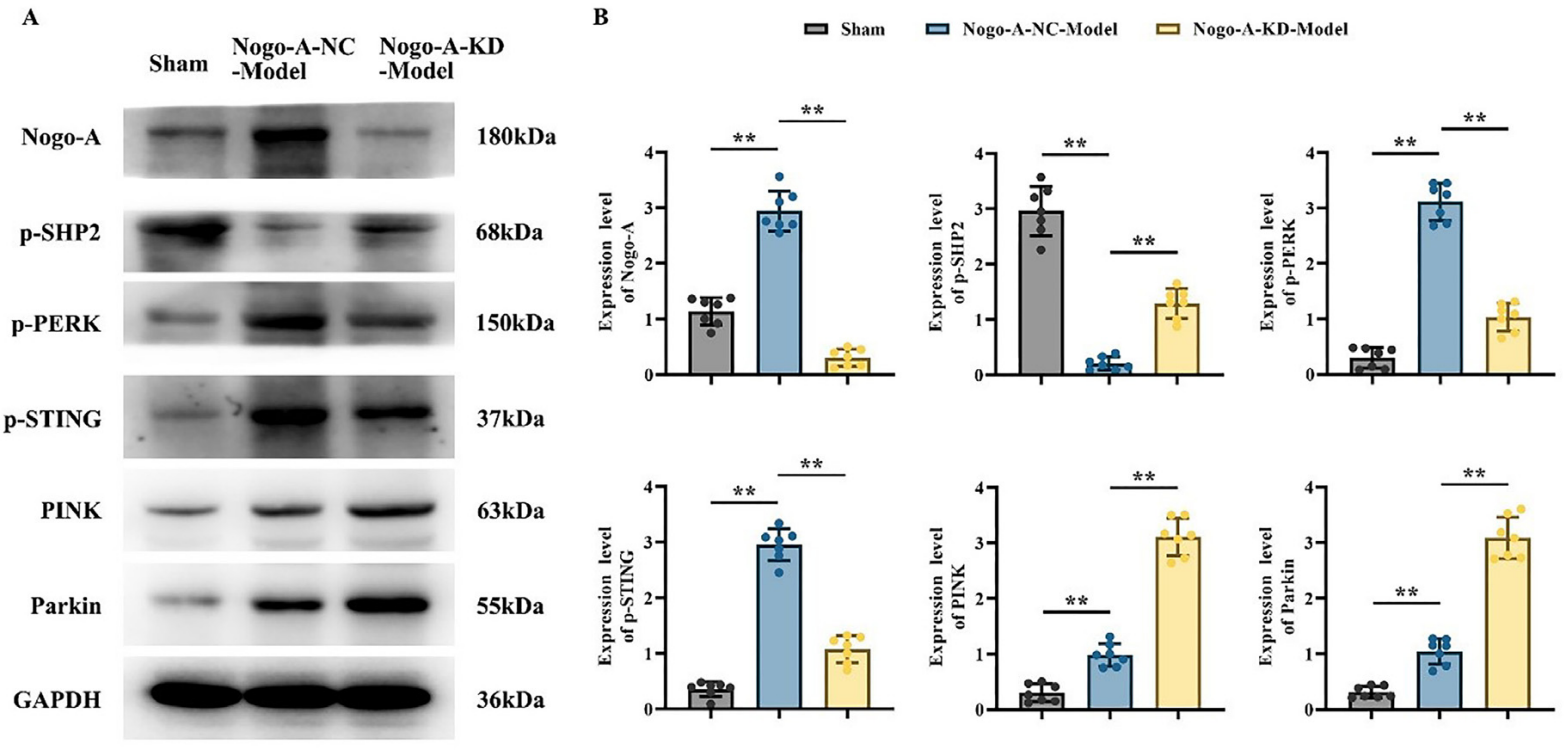
**Western blot analysis of Nogo-A and related proteins in brain tissues of mice across different groups (*n* ═ 14).** (A) Western blot results showing the expression of Nogo-A, p-SHP2, p-PERK, p-STING, PINK, and Parkin proteins in the Sham, Nogo-A-NC-Model, and Nogo-A-KD-Model groups. GAPDH was used as a loading control; (B) Quantitative analysis of Nogo-A, p-SHP2, p-PERK, p-STING, PINK, and Parkin protein expression in different groups. Compared to the Sham group, the Nogo-A-NC-Model group showed significantly increased expression of Nogo-A, PINK, Parkin, p-PERK, and p-STING proteins (*P* < 0.01), while p-SHP2 expression significantly decreased (*P* < 0.01). Compared to the Nogo-A-NC-Model group, the Nogo-A-KD-Model group showed significantly decreased expression of Nogo-A, p-PERK, and p-STING proteins (*P* < 0.01), while SHP2, PINK, and Parkin proteins were significantly increased (*P* < 0.01). These results suggest that Nogo-A can indirectly inhibit the expression of p-SHP2, PINK, and Parkin, and promote the phosphorylation of PERK and STING, thereby affecting mitochondrial autophagy. Note: Data are presented as mean ± standard deviation (Mean ± SD), ** indicates *P* < 0.01. Nogo-A-NC: Nogo-A negative control.

In the Nogo-A-NC-Model group compared to the Sham group, the expressions of Nogo-A, PINK, and Parkin were higher, with significant differences; p-PERK and p-STING expressions were higher, with clear differences; and p-SHP2 protein expression was lower, with significant differences. In the Nogo-A-KD-Model group compared to the Nogo-A-NC-Model group, the expressions of SHP2, PINK, and Parkin proteins were higher, with differences; Nogo-A protein expression was lower with significant differences; and p-PERK and p-STING protein expressions were lower, with differences (Figure 4A and 4B). The higher expressions of Nogo-A, p-PERK, and p-STING in the Nogo-A-NC-Model group compared to the Sham group, and the lower expressions in the Nogo-A-KD-Model group compared to the Nogo-A-NC-Model group, indicate that Nogo-A promotes the phosphorylation of PERK and STING. The lower p-SHP2 protein expression in the Nogo-A-NC-Model group compared to the Sham group, and the higher expression in the Nogo-A-KD-Model group compared to the Nogo-A-NC-Model group, suggest that Nogo-A indirectly inhibits p-SHP2 expression. The higher expressions of PINK and Parkin proteins in the Nogo-A-NC-Model group compared to the Sham group, and the further increase in the Nogo-A-KD-Model group, indicate that Nogo-A indirectly inhibits the expression of PINK and Parkin, thus indirectly inhibiting mitochondrial autophagy. Relative protein expression levels for the Western Blot are in the supplementary files.

### In vitro western blot experiments indicate Nogo-A inhibits SHP2 expression

**Figure 5.**
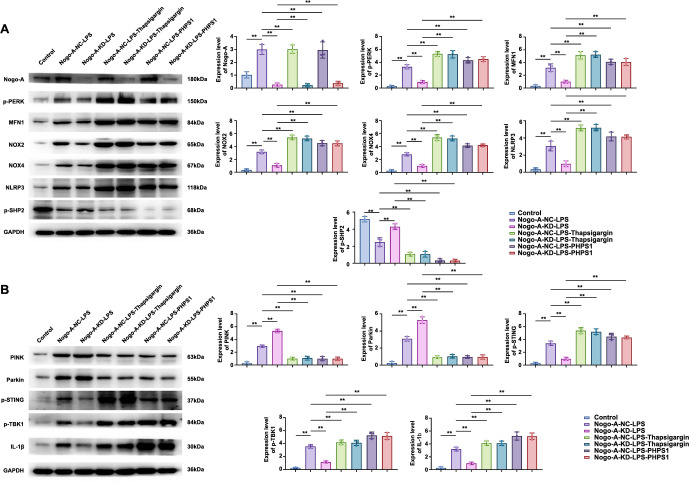
Western blot analysis of Nogo-A and related proteins in cells under different treatment conditions (*n* ═ 2*10^6^). (A) Western blot results showing the expression of Nogo-A, p-PERK, MFN1, NOX2, NOX4, NLRP3, and p-SHP2 proteins in various experimental groups. GAPDH was used as a loading control; (B) Western blot results showing the expression of PINK, Parkin, p-STING, p-TBK1, and IL-1β proteins in various experimental groups. GAPDH was used as a loading control. In Group ② compared to Group ①, Nogo-A expression was higher, with significant differences; p-PERK, MFN1, NOX2, NOX4, NLRP3, PINK, Parkin, p-STING, p-TBK1, and IL-1β expressions were high with significant differences, while other protein expressions were increased and SHP2 expression was decreased. In Group ③ compared to Group ②, Nogo-A expression was lower, with significant differences, while p-PERK, MFN1, NOX2, NOX4, NLRP3, p-STING, p-TBK1, and IL-1β protein expressions were lower, and PINK and Parkin expressions were increased along with high SHP2 expression. Groups ④ and ⑤ included the ER stress agonist: toxic carotenoid. In Group ④ compared to Group ②, Nogo-A expression remained unchanged, but p-PERK, MFN1, NOX2, NOX4, NLRP3, p-STING, p-TBK1, and IL-1β protein expressions were all higher, while SHP2, PINK, and Parkin expressions were reduced. In Group ⑤ compared to Group ③, Nogo-A expression remained unchanged, but p-PERK, MFN1, NOX2, NOX4, NLRP3, p-STING, p-TBK1, and IL-1β protein expressions were significantly increased with notable differences, while SHP2, PINK, and Parkin were reduced. Groups ⑥ and ⑦ included the SHP2 inhibitor PHPS1. In Group ⑥ compared to Group ②, Nogo-A expression remained unchanged, but p-PERK, MFN1, NOX2, NOX4, NLRP3, p-STING, p-TBK1, and IL-1β protein expressions were all higher, and SHP2 expression was significantly reduced, with PINK and Parkin both decreased. In Group ⑦ compared to Group ③, Nogo-A expression remained unchanged, but p-PERK, MFN1, NOX2, NOX4, NLRP3, p-STING, p-TBK1, and IL-1β protein expressions were significantly increased with notable differences, while SHP2 expression was significantly reduced, and PINK and Parkin were both reduced. Note: Data are presented as mean ± standard deviation (Mean ± SD), ** indicates *P* < 0.01.

BV2 cells are divided into: ① Control group (basic untreated control), ② Nogo-A-NC-LPS group (saline and LPS treatment), ③ Nogo-A-KD-LPS group (Nogo-A gene knockdown and LPS treatment), ④ Nogo-A-NC-LPS-Thapsigargin group (LPS treatment followed by Thapsigargin), ⑤ Nogo-A-KD-LPS-Thapsigargin group (Nogo-A gene knockdown and LPS treatment followed by Thapsigargin), ⑥ Nogo-A-NC-LPS-PHPS1 group (LPS treatment followed by PHPS1), and ⑦ Nogo-A-KD-LPS-PHPS1 group (Nogo-A gene knockdown and LPS treatment followed by PHPS1). In the comparison between Group ② and Group ①, Nogo-A expression was higher, with significant differences; expressions of p-PERK, MFN1, NOX2, NOX4, NLRP3, PINK, Parkin, p-STING, p-TBK1, and IL-1β were higher, indicating that sepsis elevates Nogo-A expression and promotes the expression of these proteins, while SHP2 expression was lower, indicating that Nogo-A inhibits SHP2 expression. In the comparison between Group ③ and Group ②, Nogo-A expression was lower, with significant differences, while expressions of p-PERK, MFN1, NOX2, NOX4, NLRP3, p-STING, p-TBK1, and IL-1β proteins were lower, indicating that knockdown of Nogo-A reduces the expression of these proteins. PINK and Parkin increased, suggesting that Nogo-A knockdown promotes mitochondrial autophagy, while SHP2 protein expression increased, indicating that Nogo-A inhibits SHP2 expression. In Groups ④ and ⑤, with the ER stress agonist: toxic carrot, compared to Group ②, Nogo-A expression remained unchanged, while expressions of p-PERK, MFN1, NOX2, NOX4, NLRP3, p-STING, p-TBK1, and IL-1β proteins were all higher, indicating that the induction of endoplasmic reticulum oxidative stress promotes the expression of these proteins. SHP2, PINK, and Parkin decreased, indicating that endoplasmic reticulum oxidative stress inhibits SHP2 expression and mitochondrial autophagy. Compared to Group ③, Group ⑤ showed no change in Nogo-A expression, but significant increases in p-PERK, MFN1, NOX2, NOX4, NLRP3, p-STING, p-TBK1, and IL-1β protein expressions, indicating that endoplasmic reticulum oxidative stress promotes the expression of these proteins. SHP2, PINK, and Parkin decreased, indicating that endoplasmic reticulum oxidative stress inhibits SHP2 expression and mitochondrial autophagy. In Groups ⑥ and ⑦, with the SHP2 inhibitor PHPS1, compared to Group ②, Nogo-A expression remained unchanged, but expressions of p-PERK, MFN1, NOX2, NOX4, NLRP3, p-STING, p-TBK1, and IL-1β proteins were all higher, indicating that inhibiting SHP2 promotes the expression of these proteins, while SHP2 protein expression significantly decreased, and PINK and Parkin decreased, suggesting that SHP2 promotes PINK and Parkin protein expression. Compared to Group ③, Group ⑦ showed no change in Nogo-A expression, but significant increases in p-PERK, MFN1, NOX2, NOX4, NLRP3, p-STING, p-TBK1, and IL-1β protein expressions, indicating that inhibiting SHP2 promotes the expression of these proteins. SHP2 protein expression significantly decreased, and PINK and Parkin decreased, suggesting that SHP2 promotes PINK and Parkin protein expression. Groups ④ and ⑤ had higher SHP2 protein expression than Groups ⑥ and ⑦, indicating that endoplasmic reticulum oxidative stress has a weaker capacity to inhibit SHP2 compared to Groups ⑥ and ⑦ (Figure 5A and 5B). Relative protein expression levels for the Western Blot are in the supplementary files.

**Figure 6.**
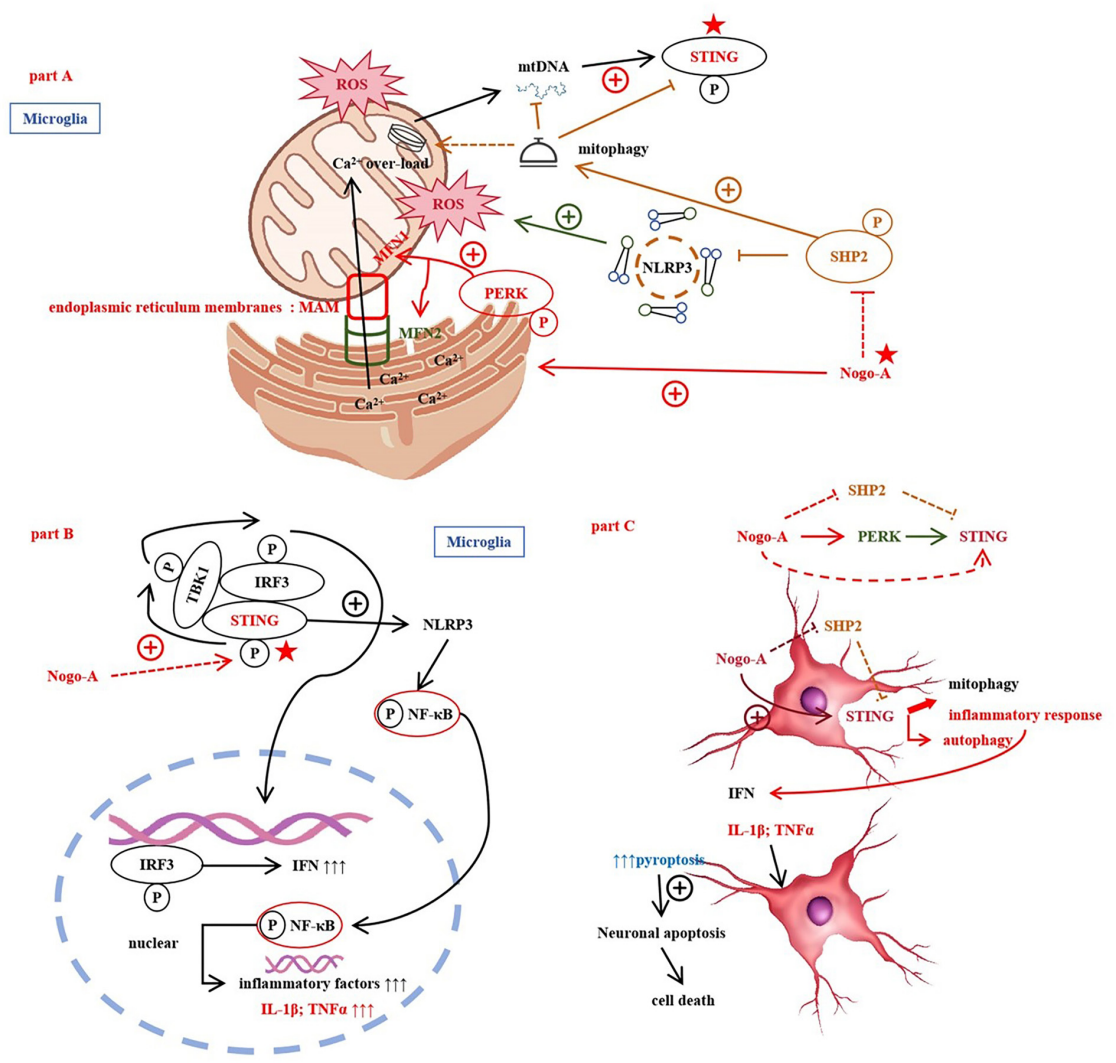
**In SAE, Nogo-A plays a critical role through a series of complex signaling pathways.** Firstly, Nogo-A regulates the endoplasmic reticulum (ER) and MAM, leading to the release of calcium ions (Ca^2+^) from the ER, which in turn causes mitochondrial overload. This overload induces the generation of reactive oxygen species (ROS). Increased ROS levels promote the activation of the NLRP3 inflammasome and affect mitochondrial autophagy (mitophagy) by activating STING (stimulator of interferon genes) and modulating the phosphorylation status of PERK (PKR-like ER kinase) and SHP2 (protein tyrosine phosphatase 2). In this process, STING activation further triggers the phosphorylation of IRF3 (interferon regulatory factor 3) and NF-κB (nuclear factor kappa B), enhancing the production of inflammatory factors such as interferon (IFN), IL-1β, and TNFα. The increase in these inflammatory factors leads to the M1 polarization of microglia, further exacerbating the neuroinflammatory response. In summary, Nogo-A exacerbates SAE by inhibiting SHP2 and activating ROS, PERK, and STING, which results in ER stress, aberrant mitochondrial autophagy, and intensified neuroinflammatory responses. SAE: Sepsis-associated encephalopathy; MAM: mitochondrial-associated membrane.

## Discussion

The mitochondria-associated endoplasmic reticulum membranes (MAM) are specialized membrane regions between mitochondria and the endoplasmic reticulum (ER), involved in calcium exchange, lipid transfer, and metabolism [11]. Calcium ions (Ca^2+^) are released from the ER and transferred to mitochondria via MAM [32]. In cardiomyocytes, silencing Nogo-A can significantly alleviate apoptosis caused by hypoxia/reoxygenation, such as DNA fragmentation and phosphatidylserine translocation. The mechanism involves maintaining mitochondrial membrane potential, inhibiting ROS accumulation, and improving intracellular calcium regulation [33]. The ER is related to maintaining protein homeostasis and is highly sensitive to changes in the cellular microenvironment [34]. In this study, Nogo-A promotes ER oxidative stress, and MAM can serve as a platform for inflammatory signaling, participating in the recruitment and release of NLRP3 and the occurrence of pyroptosis [17].

Research reports indicate that the NLRP3 inflammasome can be activated by various stimuli, including potassium ion efflux, mitochondrial dysfunction, lysosomal disruption, and degradation across the Golgi apparatus [12]. SHP phosphatases can inhibit the activity of the NLRP3 inflammasome, thereby reducing the detrimental effects of neuroinflammation [35]. Studies also show that ER stress can activate the NLRP3 inflammasome in peripheral and central immune cells. Activation of the NLRP3 inflammasome by ER stress occurs in monocytes via a calcium-dependent mechanism and an ROS-independent mechanism, with MAMs playing a crucial role in the innate immune response to ER stress [36]. PERK regulates oxidative stress in MAMs [37]. PERK also increases the DNA-binding activity of NF-κB and subsequent TNFα expression [37]. MAMs are also critical sites for initiating autophagy, removing defective organelles and misfolded proteins through specific regulatory proteins [37]. Research has further shown that mitochondrial damage and mitochondrial DNA (mtDNA) release into the cytoplasm activate the STING-IRF3 pathway [38]. Convincing evidence suggests that SHP-2 is also associated with immune signaling and inflammatory responses; removal or inhibition of SHP2 in microglia leads to enhanced NLRP3 activation and excessive production of the proinflammatory cytokine IL-1, indicating that under normal conditions, it can modulate inflammation and control tissue damage [15].

In LPS-induced cardiac dysfunction, the STING-IRF3 pathway regulates apoptosis and inflammation in cardiomyocytes by activating NLRP3 [39]. Recent studies show that PERK can interact with STING [40]. STING is a key signaling molecule in immunity and inflammation, activated by various stress signals, such as viral infections, ER stress, or mtDNA leakage into the cytoplasm [41, 42]. The STING pathway and its induced autophagy initiate effective immune defense responses upon recognizing pathogen DNA [43]. Upon activation, STING binds to kinase 1 (TBK1) and transcription factor IRF3, promoting IRF3 phosphorylation and TBK1 activation [44, 45]. After cGAMP recognizes STING, it triggers the activation of transcription factors IRF3 and NFκB and also induces inflammation through genes like type I interferon IFNβ, while modulating IRF3 transcription independently [46–48]. Recent research shows that PERK is an important component of MAMs, and its activation can alter the structure of MAMs, leading to apoptosis [49]. PERK kinase communicates with mitochondria and regulates mitochondrial-related oxidative stress [49]. Potassium efflux-induced mtDNA release activates the NLRP3 inflammasome [50].

STING (stimulator of interferon genes) is a transmembrane protein located on the outer membrane of the ER [51]. STING is a resting ER-resident protein consisting of 378 amino acids. The C-terminal domain of STING recruits and activates the TBK1 signaling pathway [52, 53]. After stimulation, STING is transported to the Golgi apparatus and the ER-Golgi intermediate compartment, where TBK1 is recruited and activates the STING signaling pathway, including the phosphorylation of STING and IRF3 [54, 55]. STING activation interferes with lysosomal acidification in an interferon-dependent manner but does not affect autophagy biosynthesis or fusion, exacerbating sepsis [43].

Our report indicates that in the presence of Signal 1 (NF-κB), the NLRP3 inflammasome is activated through mitochondrial apoptosis signaling, thereby promoting the production of IL-1β. Secondary signal activators of NLRP3 (e.g., ATP) induce mitochondrial dysfunction and apoptosis, leading to the release of oxidized mtDNA into the cytoplasm, where it binds to the NLRP3 inflammasome and activates its function [56]. Activation of the NLRP3 inflammasome requires triggering by microbial LPS, which upregulates the transcription of pro-inflammatory cytokines and inflammasome components by activating the transcription factor NF-κB [15, 57, 58]. Inflammasomes can sense various stimuli, including microbial products or other possible damage within the cytoplasm. After inflammasome assembly, Caspase 1 precursors are processed by adjacent active Caspase 1, inducing the maturation of IL-1β precursors into their activated form [13]. The activation of MAPK is also involved in high glucose-induced microglial inflammation [16]. Studies show that PERK phosphorylation induces ER stress after traumatic brain injury (TBI), and blocking PERK can rescue synaptic damage and memory deficits [59]. ER stress involves the activation of the interferon gene STING, which promotes IRF3 phosphorylation and TBK1 activation [60] Figure 6.

In this experiment, we successfully established both in vivo and in vitro sepsis models. The in vivo experiment involved creating a mouse sepsis model, with a Sham group as a blank control and a Nogo-A-NC-Model group as a negative control. The Nogo-A-NC-Model group received Nogo-A gene transfection but did not knock out the Nogo-A gene, ensuring that the observed reduction in intelligence and damage to the CA region of the hippocampus in mice was due to the loss or reduction of the Nogo-A gene, not other factors during the transfection process. Through bioinformatics analysis of the GSE135838 dataset, we identified significant expression changes in the inflammation-related gene Nogo-A (RTN4) and revealed its co-expression relationships with key genes, such as STING1, TBK1, IRF3, ROS1, and PTPN11 [61, 62]. GO and KEGG pathway enrichment analysis highlighted the expression patterns of these genes under inflammatory conditions, particularly emphasizing changes in transcriptional regulation, signal transduction, and cell adhesion, as well as the importance of the Ras, PI3K-Akt, and MAPK signaling pathways in inflammation. These results provide new insights into the mechanisms of inflammation. To further explore protein correlations, we retrieved datasets related to SAE from the GEO database and collected SAE gene expression data for normalization. DEG expression analysis identified genes significantly upregulated or downregulated between the inflammatory group and the control group. GO and KEGG analysis further revealed significant enrichment of these genes in biological processes and pathways related to neuroinflammation and immune regulation. Finally, we constructed a gene association network, identified key regulatory nodes and modules, and analyzed the correlations between SHP-2/NLRP3, ROS production, and M1 polarization. In this experiment, we also successfully established an in vitro sepsis model and divided logarithmic phase BV-2 cells into seven groups to explore the effects of Nogo-A on the expression of various proteins in microglia and to evaluate the effects of the ER stress agonist Thapsigargin and the SHP2 inhibitor PHPS1. The groups were as follows: ① Control Group (basic untreated control), ② Nogo-A-NC-LPS group (saline and LPS treatment), ③ Nogo-A-KD-LPS group (Nogo-A gene knockdown and LPS treatment), ④ Nogo-A-NC-LPS-Thapsigargin group (LPS treatment followed by Thapsigargin), ⑤ Nogo-A-KD-LPS-Thapsigargin group (Nogo-A gene knockdown and LPS treatment followed by Thapsigargin), ⑥ Nogo-A-NC-LPS-PHPS1 group (LPS treatment followed by PHPS1), and ⑦ Nogo-A-KD-LPS-PHPS1 group (Nogo-A gene knockdown and LPS treatment followed by PHPS1). Western blot analysis revealed that in the LPS-induced sepsis model (Group ②), Nogo-A expression was significantly elevated, while the expression of proteins such as p-PERK, MFN1, NOX2, NOX4, NLRP3, p-STING, p-TBK1, and IL-1β also significantly increased, and SHP2 expression decreased. This indicates that Nogo-A promotes the expression of these proteins and inhibits SHP2. In the Nogo-A knockdown condition (Group ③), Nogo-A expression significantly decreased, accompanied by a notable reduction in the expression of proteins, such as p-PERK, MFN1, NOX2, NOX4, NLRP3, p-STING, p-TBK1, and IL-1β, while PINK and Parkin expressions increased, and SHP2 expression increased. This suggests that the absence of Nogo-A inhibits the expression of proteins related to inflammation and apoptosis while enhancing mitochondrial autophagy and SHP2 expression. After adding Thapsigargin to Groups ④ and ⑤, Nogo-A levels were maintained, and the expression of related proteins such as p-PERK significantly increased, while SHP2, PINK, and Parkin levels decreased. This confirms that ER stress promotes the expression of these proteins and inhibits mitochondrial autophagy and SHP2. In groups treated with PHPS1 (Groups ⑥ and ⑦), Nogo-A expression remained unchanged, p-PERK and related proteins significantly increased, and SHP2, PINK, and Parkin levels decreased, further confirming that inhibiting SHP2 can significantly promote the expression of these proteins. Notably, SHP2 expression was higher in cells treated with Thapsigargin (Groups ④ and ⑤) compared to those treated with PHPS1 (Groups ⑥ and ⑦), indicating that ER stress has a weaker inhibitory effect on SHP2 than direct SHP2 inhibition. These results reveal the critical role of Nogo-A in regulating the expression of various important proteins in microglia and the effects of ER stress agonists and SHP2 inhibitors on these regulatory mechanisms.

Nogo-A activates the NLRP3 inflammasome by promoting ER oxidative stress and inhibiting SHP2, thereby enhancing the formation of activated caspase-1 and leading to neuronal apoptosis [16]. This mechanism involves the regulation of multiple molecules and signaling pathways, further elucidating the important role of Nogo-A in central nervous system neuroinflammation and neuroprotection. Our study provides a new theoretical basis for the treatment of SAE. Nogo-A regulates inflammatory responses and neuronal apoptosis through various signaling pathways, especially by modulating oxidative stress and mitochondrial autophagy via the ER–mitochondria axis, thereby activating the NLRP3 inflammasome. This study explores the mechanism of Nogo-A in SAE and offers new perspectives for future clinical interventions.

Although this study reveals the key role of Nogo-A in regulating ER stress, mitochondrial autophagy, and NLRP3 inflammasome activation, there are still some limitations. First, the in vivo and in vitro sepsis models used may not fully replicate the complexity of human SAE, with differences in certain physiological and pathological mechanisms between mice and humans. Second, gene knockout technology may have off-target effects that affect the accuracy of experimental results; despite using control groups, further verification of gene knockout specificity is needed. Additionally, the study primarily focuses on the role of Nogo-A, and single-gene studies may not comprehensively reveal the pathological mechanisms of the disease. Finally, the study was conducted at specific time points and in specific cell types, but the dynamic changes in ER stress and mitochondrial function may require longer and more diverse observations.

To further understand the role of Nogo-A in SAE, future research can expand in the following aspects: comprehensive studies involving multiple genes and pathways; exploring the dynamic changes of ER stress and mitochondrial function through time-series and multitissue analysis; and validating experimental results in clinical samples. Additionally, research should focus on the development of gene therapy and drug intervention strategies based on Nogo-A and its regulatory pathways, as well as an in-depth investigation of interactions between Nogo-A and other key molecules. Using single-cell sequencing and spatial transcriptomics, CRISPR/Cas9 gene editing technology, more diverse animal models, and novel imaging techniques to conduct high-throughput drug screening and long-term follow-up studies will also be beneficial. Integrating multidimensional data to construct a comprehensive molecular mechanism network for SAE will provide more effective strategies and theoretical foundations for treatment.

## Conclusion

SAE is a brain dysfunction arising from systemic inflammation, characterized by blood–brain barrier disruption, neuroinflammation, and neuronal death. This paper highlights Nogo-A’s crucial role in SAE, focusing on its impact on mitochondrial autophagy and neuronal survival by inhibiting SHP2 and activating ROS. It demonstrates that Nogo-A is central not only in neurodamage but also in modulating oxidative stress and mitochondrial autophagy via the ER-mitochondria axis, thus activating the NLRP3 inflammasome. These findings offer a new perspective on SAE pathogenesis and expand the understanding of Nogo-A’s role in neuroinflammation. While promising, the study faces limitations, such as model discrepancies, off-target effects of gene knockdown, and insufficient dynamic observations. Future research should use a multigene and multipathway approach, time-series and multitissue analysis, and clinical validation to further explore ER stress and mitochondrial function dynamics. Advancing gene therapy and drug intervention, utilizing single-cell sequencing and high-throughput screening, and constructing a detailed molecular mechanism network will enhance SAE understanding and treatment, with significant theoretical and practical implications.

## Supplemental data

Supplementary data is available at the following link: https://www.bjbms.org/ojs/index.php/bjbms/article/view/10822/3439

## Data Availability

The datasets generated during and/or analyzed during the current study are available from the corresponding author on reasonable request.
